# Unmasking Coronary Microvascular Dysfunction in Hypertrophic Cardiomyopathy: Multimodality Imaging With Stress Cardiac MRI and Coronary CT Angiography

**DOI:** 10.1155/crra/6238939

**Published:** 2026-07-08

**Authors:** Furkan Ufuk

**Affiliations:** ^1^ Department of Radiology, University of Chicago Medicine, Chicago, Illinois, USA, uchicago.edu

## Abstract

Coronary microvascular dysfunction (CMD) is a hallmark feature in hypertrophic cardiomyopathy (HCM), linked to significant clinical challenges including myocardial ischemia, fibrosis, and an elevated risk of heart failure and sudden cardiac death. In this case, a 21‐year‐old female presented with intermittent lower extremity edema, exertional dyspnea, and persistent fatigue. Imaging studies were pivotal in identifying the underlying CMD and confirming the HCM diagnosis. Transthoracic echocardiography revealed mild left ventricular hypertrophy with hyperdynamic function, reduced global longitudinal strain, and mildly increased LVOT gradients. Cardiac magnetic resonance imaging (CMR) provided more definitive findings: severe asymmetrical left ventricular hypertrophy, most pronounced in the apical inferoseptal wall, alongside subendocardial perfusion defects identified during regadenoson stress perfusion imaging. These perfusion abnormalities involved multiple myocardial segments and did not conform to a single epicardial coronary artery distribution, supporting a microvascular pattern of ischemia and strongly implicating CMD as the underlying cause of myocardial ischemia. The absence of late gadolinium enhancement, coupled with elevated native T1 and extracellular volume values, suggested early interstitial expansion without extensive fibrosis. Coronary computed tomography angiography confirmed the absence of significant epicardial coronary artery disease, further substantiating that the ischemia was driven by microvascular dysfunction rather than large‐vessel obstruction. Structural abnormalities in the coronary microvasculature, such as intimal and medial hyperplasia, reduced lumen size, and impaired vasodilatory capacity, were likely contributors to the CMD observed in this patient. In summary, this case highlights the crucial role of advanced imaging techniques, such as CMR stress perfusion and T1 mapping, in diagnosing CMD within the context of HCM. These findings underscore the need for tailored management strategies targeting CMD to mitigate its impact on clinical outcomes in HCM patients.

## 1. Introduction

Hypertrophic cardiomyopathy (HCM), a genetic cardiac disorder, is characterized by an abnormal thickening of the heart′s muscular walls and affects approximately one in every 500 individuals. A pivotal contributor to the clinical burden of HCM is coronary microvascular dysfunction (CMD), a pathophysiological process in which structural abnormalities, such as intimal and medial hyperplasia, narrow the coronary microvascular lumen and impair blood flow [1]. These microvascular changes lead to inadequate myocardial perfusion, resulting in chronic ischemia. Over time, the sustained ischemic environment can trigger myocardial fibrosis, promoting adverse left ventricular remodeling and functional decline. CMD not only exacerbates HCM′s progression but also significantly elevates the risk of heart failure and sudden cardiac death [1, 2]. As such, understanding CMD′s role in the disease process is crucial for improving patient outcomes and developing targeted therapeutic strategies.

## 2. Case Presentation

A 21‐year‐old woman presented with intermittent lower extremity edema and exertional dyspnea. Her medical history was notable for Type 2 diabetes mellitus managed with metformin, morbid obesity (BMI 37.5 kg/m^2^), untreated obstructive sleep apnea (OSA) due to CPAP intolerance, and recently diagnosed hypertension for which she was prescribed losartan. Her family history included significant cardiovascular and metabolic conditions, with maternal and paternal relatives affected by hypertension, diabetes, and stroke. She denied chest pain but reported persistent fatigue and morning tiredness. A routine ECG, performed during an emergency room visit, showed nonspecific ST‐T wave changes, prompting her referral to cardiology. On physical examination, she was normotensive (BP 137/88 mmHg) with a heart rate of 82 bpm. Cardiovascular examination revealed no murmurs, gallops, or rubs, and lower extremity pulses were intact without evidence of edema. Laboratory tests showed mild elevation of BNP (106 pg/mL; normal range < 100 pg/mL), hyperglycemia, hypertriglyceridemia (261 mg/dL; normal range < 150 mg/dL), and low HDL (31 mg/dL; normal range > 40 mg/dL).

Transthoracic echocardiography demonstrated mild left ventricular hypertrophy with hyperdynamic systolic function (EF > 75%) and reduced global longitudinal strain (−6.6%), predominantly in the apical segments. There were mild increases in left ventricular outflow tract (LVOT) gradients (19 mmHg with Valsalva). Given these findings, cardiac magnetic resonance imaging (CMR) with regadenoson stress perfusion was performed. CMR revealed asymmetrical left ventricular hypertrophy, most prominent in the apical inferoseptal wall (15 mm), suggestive of HCM (Figure 1). Rest perfusion imaging was unremarkable. However, regadenoson stress perfusion imaging demonstrated diffuse subendocardial perfusion defects across multiple segments and did not conform to a single epicardial coronary artery distribution, indicating CMD rather than epicardial coronary artery disease (Figure 2). Late gadolinium enhancement (LGE) imaging was unremarkable, but T1 mapping revealed increased native T1 values (1202 ms; normal range 1050–1200 ms), and the extracellular volume (ECV) was mildly elevated at 34%, suggesting early interstitial expansion. Coronary CT angiography confirmed no significant epicardial coronary artery disease (Figure 3).

**Figure 1:**
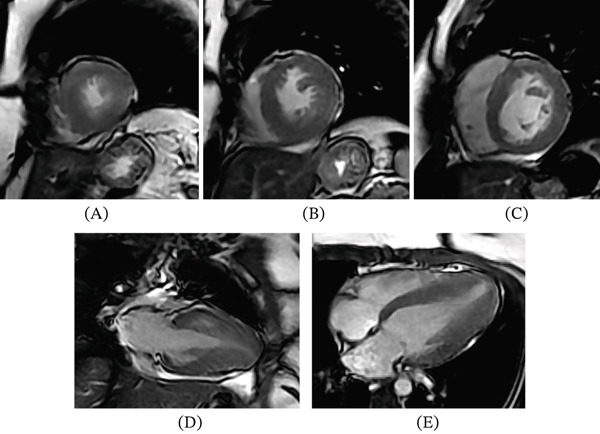
(A–C) Short axis, (D) two‐chamber view, and (E) four‐chamber view balanced steady‐state free precession cine acquisitions at end‐diastolic phase from a 3 T MRI scanner. MR images show apical and mid‐left ventricular hypertrophy.

**Figure 2:**
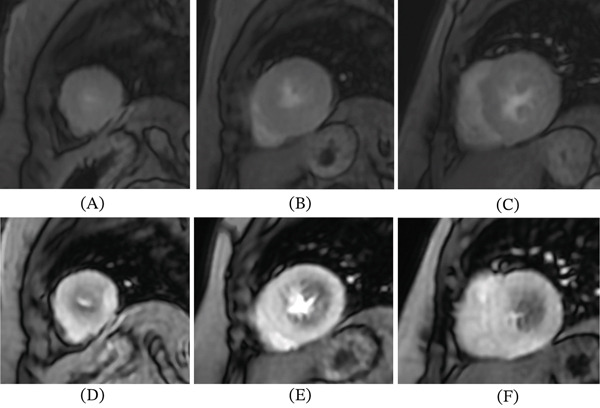
(A–C) Dynamic postcontrast cardiac MR (CMR) images at rest and (D–F) following pharmacologic stress with the vasodilator regadenoson. (D–F)The CMR stress perfusion images with regadenoson reveal diffuse subendocardial perfusion defects across the apex, mid, and basal lateral and inferior walls (arrows).

**Figure 3:**
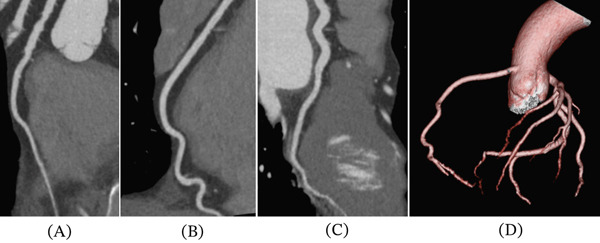
Coronary CT angiography (CCTA) images of the (A) left anterior descending (LAD) coronary artery, (B) left circumflex (LCx) coronary artery, and (C) right coronary artery reveal no evidence of coronary artery stenosis or occlusion. (D) The three‐dimensional volume‐rendered image demonstrates no evidence of coronary artery stenosis.

The patient′s treatment strategy focused on controlling cardiovascular risk factors and alleviating symptoms. Beta‐blockers were introduced to reduce myocardial oxygen demand and improve perfusion, and losartan therapy was continued for hypertension. Emphasis was placed on lifestyle modifications, including weight loss and dietary improvements. She was referred to a sleep specialist to reconsider CPAP therapy, given her untreated OSA and associated cardiovascular risk. Long‐term follow‐up was planned to monitor symptom progression, cardiac function, and potential myocardial fibrosis. Future considerations include exploring pharmacologic options targeting CMD, such as ranolazine or sodium‐glucose cotransporter‐2 (SGLT2) inhibitors, which may enhance myocardial energetics.

## 3. Discussion

CMD is a key pathological feature in HCM, contributing to myocardial ischemia, fibrosis, and adverse cardiovascular outcomes. On cardiac MRI, elevated native T1 and mildly increased ECV values are indicative of early interstitial remodeling and diffuse myocardial changes in HCM. Importantly, these findings occur in the absence of LGE, which suggests that T1 mapping techniques can detect subtle myocardial involvement prior to the development of overt fibrosis on conventional LGE imaging, as in the present case [3].

CMD in HCM is primarily attributed to structural abnormalities of the intramyocardial arterioles, such as intimal and medial hyperplasia, reduced lumen diameter, and diminished vasodilatory reserve. These changes compromise myocardial perfusion during stress, even in the absence of obstructive epicardial coronary artery disease, resulting in chronic ischemia, myocardial remodeling, and subsequent fibrosis [1–3]. CMD has significant prognostic implications in HCM and is associated with progressive myocardial fibrosis, ventricular remodeling, arrhythmias, heart failure progression, and adverse cardiovascular outcomes. Chronic microvascular ischemia may facilitate disease progression and elevate the risk of sudden cardiac death, highlighting the necessity for early detection and ongoing longitudinal monitoring [3–6].

Kutty et al. [4] demonstrated that CMD detected through myocardial contrast echocardiography is linked to adverse clinical outcomes, including increased mortality, even in the absence of epicardial coronary artery disease. CMD is also implicated in other conditions, including Anderson–Fabry disease, where it contributes to severe left ventricular dysfunction despite normal coronary arteries. Amigues et al. [5] further reported that CMD is prevalent in rheumatoid arthritis patients, linking systemic inflammation to microvascular impairment and an increased cardiovascular risk. Given the lack of standardized treatment guidelines, current management strategies focus on optimizing hemodynamics, reducing myocardial oxygen demand, and controlling associated risk factors. Beta‐blockers and calcium channel blockers are commonly used, whereas experimental therapies, including novel vasodilators and metabolic modulators, remain under investigation. Expanding our understanding of CMD across various conditions emphasizes the need for targeted diagnostic strategies and tailored treatments to improve patient outcomes [5–8].

In conclusion, CMD is a critical determinant of adverse outcomes in HCM, contributing to myocardial ischemia, fibrosis, and progression to heart failure. Advanced imaging modalities have enhanced our ability to detect CMD, facilitating early intervention. Recognizing CMD′s role across various cardiovascular conditions underscores the need for tailored diagnostic and therapeutic approaches to improve patient outcomes.

## Funding

No funding was received for this manuscript.

## Consent

No written consent has been obtained from the patient as there is no patient identifiable data included in this case report/series.

## Conflicts of Interest

The author declares no conflicts of interest.

## Data Availability

Data sharing is not applicable to this article as no datasets were generated or analyzed during the current study.
